# Bias Mitigation Across the Healthcare Artificial Intelligence Lifecycle: A Structured Narrative Review

**DOI:** 10.7759/cureus.116223

**Published:** 2026-09-14

**Authors:** Wenzhi Tseng, Kyla S Holbrook, Radhika L Edpuganti, Mariam Girgis, Dana Chen, Benson Babu

**Affiliations:** 1 Internal Medicine, New York Medical College, New York, USA; 2 Internal Medicine, New York Medical College, Valhalla, USA; 3 Internal Medicine, Henry Ford Genesys, Grand Blanc, USA; 4 Internal Medicine, Mount Sinai Medical Center, Miami, USA; 5 School of Osteopathic Medicine, A.T. Still University, Mesa, USA; 6 Internal Medicine, Wyckoff Heights Medical Center, New York, USA

**Keywords:** artificial intelligence in medicine, bias in healthcare, bias mitigation, performance metrics, sampling method

## Abstract

Bias remains a difficult issue when applying artificial intelligence (AI) in healthcare. Many types of bias can appear at various stages of AI development, and bias has been shown to perpetuate disparities in vulnerable populations. Current literature tends to either be an exploration of a particular method or a review discussing multiple approaches but fails to explore how the approaches work together. This narrative review summarizes recent approaches to mitigating bias in healthcare AI and organizes them within a practical lifecycle framework spanning data acquisition, preprocessing, model training, validation, and deployment.

A structured literature search was conducted using PRISMA-informed principles. Keywords “Artificial intelligence”, “bias mitigation”, and “artificial intelligence bias mitigation” were searched on PubMed, Embase, Web of Science, Semantic Scholar, MedRxiv, and Google Scholar. Articles from 2018 to 2024 in English that explained a method for mitigating bias in AI were included. Reviews were excluded. The search identified 340 records. After removal of duplicates and screening, 16 articles were included.

Many techniques were designed to guide the composition of the training data, mainly sampling methods. Datasets are also augmented with newer methods such as adversarial learning and subpopulation techniques. New model structures, loss functions, and algorithms are being applied in medical settings. The review explains the mechanics behind these methods and the settings in which they can be used.

Addressing AI bias in healthcare requires multiple approaches targeting many stages of AI development, including data preprocessing, model training, and validation. Recent innovations include adversarial learning and hybrid models. We propose a practical lifecycle protocol encouraging multifaceted approaches that address bias at all stages of development. Ongoing studies are needed to validate these methods clinically.

## Introduction and background

Introduction

The use of artificial intelligence (AI) in medicine is still relatively nascent, despite the many recent advancements in theory and usage in other fields. One of the main issues when using AI in medicine is bias against certain subpopulations. AI bias may manifest not only as unequal model performance across subpopulations but also as unequal calibration, unequal false-positive or false-negative rates, differential missingness, or reduced generalizability across institutions [1]. Commonly cited examples include AI programs demonstrating poorer performance against minority races or ethnicities [2]. The stages of AI development are generally categorized into data acquisition, data preprocessing, model training, model evaluation or validation, and deployment [3]. Bias can appear at each of these stages. At the level of data collection, there may be fewer samples from a certain subpopulation, limiting the model’s performance for that subpopulation. At the level of deployment, bias can occur at the level of human evaluators if the model is applied inappropriately or misinterpreted. Researchers must be wary of bias occurring against any number of subpopulations at any stage of AI development, making bias mitigation a very difficult problem [1].

The current literature has demonstrated the need for bias mitigation and proposed numerous solutions, but many papers focus on one particular problem or method and do not provide insight on how their tools may be generalized. Current reviews frequently describe isolated fairness techniques without integrating them into a clinically actionable framework. Reviews of AI bias mitigation techniques often shy away from the mechanistic detail behind statistical tools and algorithms and often do not address the subtleties behind choosing performance metrics. To address these gaps, we present a qualitative evidence review with the aim to summarize recent approaches to AI bias mitigation in healthcare, explain the mechanisms behind commonly used methods, and propose a practical lifecycle framework for applying these approaches across stages of clinical AI development.

Methods

A structured literature search was conducted using PRISMA-informed principles to improve transparency in article identification and selection. The databases searched were PubMed, MedRxiv, Semantic Scholar, Web of Science, Embase, and Google Scholar. Search terms and filters are provided in Table 1. Only articles in English, published between January 1, 2018, and October 24, 2024, that presented and explained a method for mitigating bias in AI were included. The database search was conducted on October 24, 2024. The search entries were each reviewed independently by two reviewers. Only full length articles were included. Peer-reviewed articles were prioritized. Preprints were considered only when they described a relevant method not otherwise represented in the peer-reviewed literature, search terms for MedRxiv were therefore searched in the abstract and title as MedRxiv is comprised entirely of preprints. Studies using MIMIC data were excluded if their primary focus was technical benchmarking rather than clinically contextualized bias mitigation. This is because MIMIC data are deidentified archival data and not real-time patient data, and therefore some studies using MIMIC data lacked sufficient clinical application to fall within the scope of this investigation. The search identified 340 records.

**Table 1:** Article screening search terms

Database	Search terms	Filters
Pubmed	((artificial intelligence) AND (bias mitigation) AND ((healthcare) OR (clinical))) AND (("2018"[Date - Publication] : "2024"[Date - Publication]))	Full Text, English, Humans, No Reviews, No Systematic Reviews
MedRxiv	artificial intelligence bias	Posted between “01 Jan, 2018 and 24 Oct, 2024”
Semantic Scholar	artificial intelligence mitigate bias mitigation in medicine medical clinical healthcare	Date Range 2018-2024, Field of Study = Medicine, Has PDF
Web of Science	artificial intelligence bias mitigation medicine	English, Publication Year 2018-2024
Embase	('artificial intelligence'/exp OR 'artificial intelligence') AND ('bias mitigation'/exp OR 'bias mitigation' OR 'mitigate bias') AND ('medical' OR 'medicine'/exp OR 'medicine' OR 'clinical'/exp OR 'clinical' OR 'healthcare'/exp OR 'healthcare')	Only in English, Humans, Publication Year 2018-2024,
Google Scholar	"artificial intelligence" and ("bias mitigation" or "mitigate bias") medicine,medical,clinical,healthcare	Dated between 2018-2024, without “systematic review”

There were five reviewers total. If two reviewers disagreed on the inclusion of an article, the project leader reviewed the article and made the final decision. After removal of duplicates and screening, articles were independently reviewed by the project leader and principal investigator for quality. Each included study was evaluated using a standardized evidence appraisal framework developed for this review to assess both methodological rigor and clinical readiness. Eight domains were assessed: (1) overall and subgroup sample size, (2) risk of overfitting, (3) internal validation, (4) external validation using an independent institution, population, or dataset, (5) representation and analysis of clinically relevant subgroups, (6) handling of missing data, (7) assessment of model calibration, and (8) clinical applicability. Information not explicitly reported in the original study was classified as “not reported” rather than inferred. Clinical applicability was categorized as technical/model development, evaluation, retrospective clinical evaluation, independent or external clinical validation, prospective clinical evaluation, or real-world clinical implementation. Based on the totality of these domains, evidence maturity was classified descriptively as preliminary technical evidence, clinical validation evidence, or clinical implementation evidence. These categories were intended to distinguish demonstration of technical performance from evidence supporting generalizability or real-world clinical use and were not treated as a validated quantitative risk-of-bias score.

Preliminary technical evidence was defined as demonstration of a mitigation method primarily using development data, internal testing, simulated data, or a single retrospective dataset without convincing independent clinical validation. Clinical validation evidence required evaluation using clinically derived data with appropriate validation and evidence of generalizability, preferably through an independent dataset, institution, or population. Clinical implementation evidence required prospective evaluation or deployment within an actual clinical workflow with assessment of real-world performance, fairness, or clinical consequences. In total, 16 articles were included in the narrative synthesis. Inclusion and exclusion of papers is expounded in Figure 1. Evidence appraisal of the included studies is shown in Table 2.

**Figure 1:**
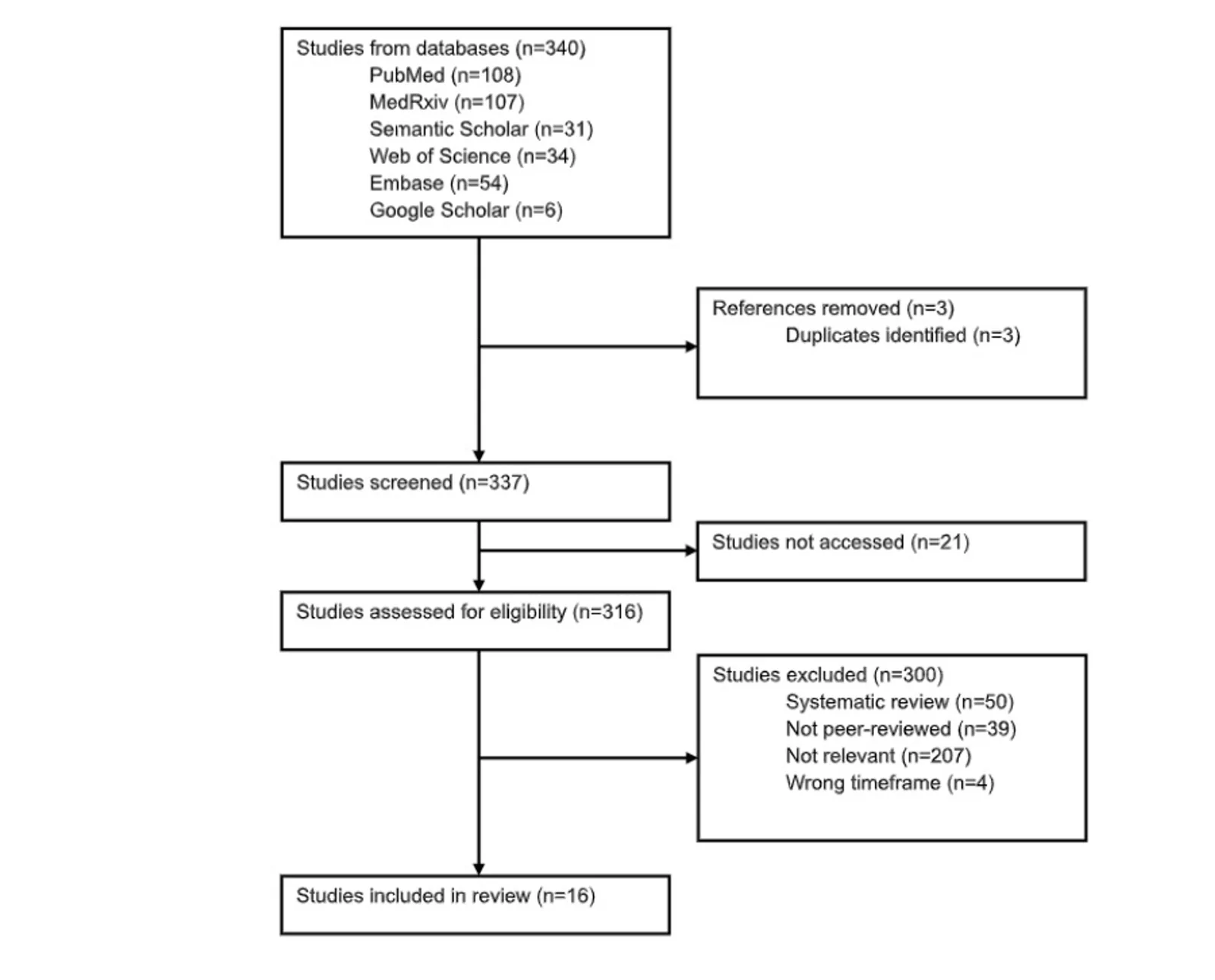
PRISMA flowchart demonstrating article inclusion and exclusion.

**Table 2:** Evidence appraisal and clinical readiness of included studies Evidence maturity reflects validation of the bias mitigation approach, not overall study quality. “Preliminary technical evidence” indicates that independent clinical generalizability or implementation remains limited. Overfitting is reported as an observable risk indicator rather than an unvalidated low/moderate/high score. CITL, calibration-in-the-large; CRC, colorectal cancer; CV, cross-validation; EHR, electronic health record; FRAX, Fracture Risk Assessment Tool; HIC, high-income country; LMIC, low- and middle-income country; MICE, multiple imputation by chained equations; NR, not reported or not clearly established from the published report; OKS, Oxford Knee Score; PCE, Pooled Cohort Equations; QA, quality assurance; RT, radiation therapy; TKR, total knee replacement; USC, University of Southern California.

Study	Sample size/data	Subgroup representation	Overfitting risk indicators	Internal validation	External validation	Missing data	Calibration	Clinical applicability	Evidence maturity
Heidari et al. [4]	329 patients; balanced dataset n=286; training n=231, reduced to 218 after 13 outliers; test n=55	Sex evaluated; original cohort 187 male/142 female; balanced to 144 male/142 female	Single cohort; downsampling reduced effective N; small clinically relevant TKR subset (n=45)	Yes: 80/20 train-test split; TKR-suitable subset also tested	No: authors explicitly state external validation is still required	All 329 completed OKS follow-up; other missingness not clearly reported	No formal probability calibration identified	Retrospective clinical prediction/development study	Preliminary technical evidence
Zhao et al. [5]	CRC microarray dataset; exact total N not clearly recoverable from the accessible report used for this audit	Class imbalance (healthy vs CRC), not demographic fairness	High-dimensional gene expression setting; feature selection and resampling used; exact events-per-feature adequacy not established	Yes: algorithmic validation/comparative testing reported	No independent clinical cohort identified	NR	No	Technical diagnostic model development	Preliminary technical evidence
Brown et al. [6]	61 acceptable + 12 erroneous RT plans; disjoint 273 acceptable plans used for feature development	Minority class = erroneous treatment plans; not demographic subgroup	Only 12 minority examples; cross-validation used	Yes: cross-validation	No independent institution/population identified	NR	No	Proof-of-principle automated radiotherapy QA	Preliminary technical evidence
Li et al. [7]	109,490 individuals	Sex and race evaluated; 64.5% female; 86.3% White; 13.7% Black	Large cohort, but single health system; resampling may repeatedly reuse relatively scarce minority-positive cases	Yes: internal model evaluation	No: single Vanderbilt University Medical Center EHR cohort	NR	No formal subgroup probability calibration emphasized	Large retrospective clinical EHR study	Preliminary technical evidence
Burlina et al. [8]	EyePACS source: 88,692 fundus images from 44,346 individuals; experimental imbalance scenario constructed	Lighter- vs darker-retinal pigmentation evaluated	Synthetic augmentation and deliberately constructed imbalance/domain shift; performance may not generalize to naturally occurring clinical imbalance	Yes: separate training/testing design	No independent external clinical cohort	NR	No formal probability calibration identified	Retrospective experimental retinal diagnostic study	Preliminary technical evidence
Franchet et al. [9]	Multicenter breast cancer histology cohorts (PACS04/05/08)	Center/stain-associated domain bias rather than protected demographic group fairness	Multicenter design and cross-center testing reduce overfitting concern relative to single-center evaluation	Yes	Yes: models trained in one center and assessed on images from other centers	NR	No formal probability calibration identified	Multicenter retrospective digital pathology validation	Clinical validation evidence
Le et al. [10]	HAM10000; 10,015 images representing 7,470 unique lesions; held-out testing reported in the study workflow	Class imbalance across seven lesion classes; not demographic fairness	Class imbalance; transfer learning, dropout/class weighting/focal loss used; no independent external dataset	Yes: internal train/validation/test evaluation	No independent external dataset	NR	No	Technical dermatologic image classification	Preliminary technical evidence
Stanley et al. [11]	Synthetic 3D brain MRI counterfactual datasets; controlled bias scenarios; multiple model seeds	Controlled simulated bias/non-bias groups; not real demographic populations	Controlled synthetic design limits clinical overfitting inference; repeated seeds support experimental stability	Yes: held-out synthetic evaluation/repeated seeds	No real-patient external validation	Not applicable to synthetic dataset	No clinically meaningful probability calibration	Controlled in-silico bias-mitigation experiment	Preliminary technical evidence
Yang et al. [12]	42,385 training; 33,371 validation; 33,095 held-out test presentations	Hospital/site representation across HIC and LMIC settings explicitly evaluated	Large multicenter datasets with separate validation and test sets	Yes	Yes: geographically/institutionally distinct hospital datasets	NR	Formal subgroup probability calibration not a central endpoint	Multicenter retrospective clinical COVID-19 screening	Clinical validation evidence
Isensee et al. [13]	BraTS challenge data with predefined validation/test evaluation	Tumor/background class imbalance; not protected demographic subgroup fairness	Extensive augmentation used to reduce overfitting; challenge-based evaluation	Yes: BraTS validation/test sets	Partial: independent challenge test set but not prospective clinical external validation	NR	Not a central endpoint	Technical medical-imaging challenge	Preliminary technical evidence
Cavallaro et al. [14]	5,190 hospital admission events; single UK health trust	Patient characteristics included; protected group fairness not the primary evaluation	125 features; cross-validation/test evaluation reported; single-institution data	Yes: cross-validation plus test evaluation	No independent external institution; authors state validation studies are necessary before extension to other cohorts	NR; paper notes missing culture information may limit use and calls for future modeling of missing variables	No formal subgroup calibration demonstrated	Retrospective clinical antimicrobial-stewardship modeling	Preliminary technical evidence
Barda et al. [15]	PCE n=1,021,041; FRAX n=1,116,324; 70/30 train-test split	Intersectional subgroups defined by age, sex, ethnicity, socioeconomic status, and immigration history; minimum subgroup size 5,000	Very large cohorts; algorithm evaluated on a test set never seen by the recalibration algorithm; bootstrap CIs	Yes: 70/30 split; test set unseen by recalibration algorithm; 200 bootstrap repetitions	No independent external institution/dataset: validation was within the same Clalit-derived populations	Yes: missing data were imputed once using MICE	Yes: CITL, calibration slope, calibration plots, Brier score; subgroup calibration directly assessed	Large retrospective clinical recalibration study	Clinical validation evidence
Dakshit et al. [16]	Three public ECG/EEG healthcare time series datasets; at least one dataset contained only 45 patients	Protected variable and combined protected group bias analyzed	Some benchmark datasets are small; framework-oriented evaluation	Yes: internal benchmark evaluation	No independent prospective clinical validation	NR	No formal clinical probability calibration identified	Framework/benchmark evaluation of healthcare time series bias	Preliminary technical evidence
Benčević et al. [17]	1,758 dermoscopic/clinical images from three datasets	Skin color evaluated using multiple quantification approaches	Multiple datasets and out-of-sample evaluation strengthen generalizability; overall imaging sample remains modest	Yes	Yes: leave-one-dataset-out/out-of-sample evaluation across three datasets	NR	NR	Multi-dataset retrospective skin lesion segmentation evaluation	Clinical validation evidence
Kiyasseh et al. [18]	Surgical video data from USC, St. Antonius Hospital, and Houston Methodist Hospital	Disadvantaged surgeon subcohorts explicitly evaluated	10-fold Monte Carlo CV; cross-hospital evaluation; some surgeon sub-cohorts remain modest	Yes: 10-fold Monte Carlo cross-validation	Yes: trained at USC and evaluated/deployed on data from two other hospitals	NR	No formal probability calibration identified	Multi-institutional retrospective surgical AI evaluation	Clinical validation evidence
Moskalenko et al. [19]	DermaMNIST primary task; BloodMNIST and PathMNIST used for adaptation; fault/adversarial tests repeated 100 times	Dataset classes and perturbation/resilience scenarios, not demographic patient subgroups	Controlled benchmark environment; repeated randomized tests; limited direct clinical generalizability	Yes: repeated benchmark testing	No real-world external clinical validation	Not clinically characterized/NR	Yes: uncertainty calibration is part of the resilience-aware MLOps framework but not subgroup clinical risk calibration	Experimental resilience/MLOps study	Preliminary technical evidence

Since the purpose of this review was to synthesize major methodological approaches rather than perform a meta-analysis or exhaustive evidence map, findings were narratively grouped according to the stage of AI development in which each mitigation strategy is most applicable. Due to the heterogeneity of the included studies spanning different mitigation techniques, performance metrics, and objectives, a quantitative meta-analysis was not conducted.

## Review

Results

Training Datasets

Key article information is summarized in Table 3. Bias frequently arises due to the composition of the training dataset. For example, a model may exhibit decreased performance on certain subgroups of the population due to the training data having fewer samples from that subgroup for the model to learn from. Some authors tackle this issue by using sampling methods to compose better training datasets; we summarize the results of these authors here and defer mechanistic detail to the discussion section. Heidari et al. attempted to predict which variables can predict response to a specific treatment for knee osteoarthritis [4]. They used downsampling and outlier removal to increase model accuracy, achieving moderate predictive accuracy. However, no comparison was done to a model without the sampling method, so little inference regarding its efficacy as a bias mitigation tool can be made. Zhao et al. used gene analysis to predict colorectal cancer [5]. They employed RUSBoost, which randomly undersamples the majority group, uses AdaBoost, and assigns larger weights to misclassified samples to boost the undersampled group. RUSBoost increased the algorithm’s sensitivity and specificity from 0.8488 +/- 0.0068 to 0.9327 +/- 0.0016 and 0.8278 +/- 0.0073 to 0.9404 +/- 0.0013, respectively.

**Table 3:** Summary of included articles †ACC1 and ACC2 are the paper’s own accuracy metrics. ASSD, average symmetric surface distance; AUPRC, area under the precision-recall curve; AUROC, area under the receiver operating characteristic curve; DSC, Dice similarity coefficient; FPR-1, false-positive rate at TPR = 1; GBT, gradient-boosted tree; HD, Hausdorff distance; MKF-SVM, mixed-kernel function support vector machine; NPV, negative-predictive value; PPV, positive-predictive value; Sens, sensitivity; Spec, specificity; TPR, true-positive rate.

Topic	Author	Year	Models used	Performance metrics
Sampling methods	Heidari et al., 2024 [4]	2024	Random forest regression	Root mean squared error (6.72), mean absolute error (5.38)
	Zhao et al., 2019 [5]	2019	MKF-SVM, WOA, RUSBoost	G-mean (0.9365), Sens (0.9327), Spec(0.9404)
	Li et al., 2023 [7]	2023	Log regression, random forest, GBT	Equal opportunity distance (0.027), disparate impact (1.148)
Training dataset construction	Brown et al., 2018 [6]	2018	SVM, SMOTE	AUROC(0.9167), FPR-1 (0.1988)
	Benčević et al., 2024 [17]	2024	U-Net, ResNet18	DSC (0.744), HD (37.342), ASSD (10.814)
Synthetic data	Burlina et al., 2021 [8]	2021	StyleGAN	Accuracy (0.7475), Sens (0.56), Spec (0.86), AUROC (0.79)
	Moskalenko et al., 2024 [19]	2024	ResNet18	ACC1 (0.747), ACC2^†^ (0.798)
	Franchet et al., 2024 [9]	2024	HEnorm, AugmentHE	Average standard deviation (0.0107), AUROC (0.84)
Model structure	Le et al., 2020 [10]	2020	ResNet50	Accuracy (0.90), precision (0.81), Sens (0.80), F1-score (0.80), AUROC (0.9863)
	Stanley et al., 2024 [11]	2024	SimBA	Relative ΔTPR (12.22%), relative ΔFPR (15.51%)
	Yang et al., 2024 [12]	2024	XGBoost	AUROC (0.876), AUPRC (0.609), Sens (0.801), Spec (0.788), NPV (0.977), PPV (0.259)
	Kiyasseh et al., 2023 [18]	2023	SAIS, TWIX	AUROC (0.866), ΔNPV (0.1)
	Isensee et al., 2018 [13]	2018	U-Net	Dice value (0.85), Sens (0.91), PPV (0.80)
	Cavallaro et al., 2023 [14]	2023	GBT with XGBoost	AUROC (0.78)
	Barda et al., 2021 [15]	2021	PCE, FRAX	Calibration-in-the-large (1.039), calibration slope (0.876)
Bias recognition	Dakshit et al., 2023 [16]	2023	BAHT	Bias score (0.88)

Brown et al. aims to judge quality assurance plans for radiation therapy for prostate cancer as either acceptable or unacceptable, with a majority of their training dataset comprising acceptable cases [6]. They combat this bias with two types of sampling methods, ensemble outlier filtering and normalized-cut sampling techniques. These methods demonstrated the strongest improvements in performance as measured by area under the curve (AUC) and false-positive rate with true positive rate equal to 1 (FPR-1). For instance, in one of their support vector machine models named RawSVM, using both ensemble outlier and normalized-cut techniques increased AUC from 0.9023 to 0.9167 and FPR-1 from 0.2226 to 0.1988, with p-values for both comparisons being less than 0.001. Li et al. aims to predict cardiovascular events such as myocardial infarction and stroke using medical health records with an eye on improving model performance on minority subgroups, including female gender [7]. They used resampling by proportion to create training datasets, which significantly improved their performance metrics, equal opportunity distance (EOD), and disparate impact (DI), for most of their models. EOD is the difference in true positive rates between the privileged and underprivileged group, given by EOD = P(Y'=1 | A=a, Y=1) - P(Y'=1 | A=a', Y=1) where Y' is the predicted label, Y is the actual label, A is protected attribute, a denotes the privileged group, and a' denotes the underprivileged group. Hence, an EOD of zero implies total fairness with respect to this metric, and a larger EOD suggests decreased fairness. DI is the ratio of the positive prediction rate in the majority group to the positive prediction rate in the minority group, given by P(Y'=1 | A=a)/P(Y'=1 | A=a') where the variables are defined the same as above. Hence, a DI of 1 implies total fairness with respect to this metric, and values away from 1 suggest decreased fairness. In Li et al's logistic regression model where the underprivileged group was female gender, EOD decreased from 0.136 to 0.027 and DI dropped from 1.587 to 1.148. However no p-values were available in their manuscript. For their deep learning model, resampling by size and proportion decreased the bias across races, but the changes were not statistically significant. 

Many papers use their datasets to create synthetic data that resemble the minority population and train their model on those synthetic images. Burlina et al. uses StyleGAN to generate synthetic images, followed by latent space manipulation to create new images with the markers of the minority population [8]. The model was then trained on these synthetic images. They also compared this to a model where more accent was put on the undersampled population. Overall accuracy increased from 66.75% (62.13, 71.37) to 74.75% (70.49, 79.01) and 71.75% (67.34, 76.16) for accented and generated data respectively, where the 95% confidence intervals are shown in brackets. However, there was no statistically significant result at the 5% level, which can be observed by overlapping confidence intervals. Franchet et al. aimed to improve H&E pathology slide identification by developing a bias-reducing algorithm called AugmentHE, which was used to improve the AI model HENorm [9]. AugmentHE, in particular, focuses on allowing the program to better identify stain color variation by modifying existing stain samples to a wider variation and colors and adding them to the training set. Every model using AugmentHE led to significant increase in AUC over those that did not use AugmentHE, with AUC increasing from 0.67 +/ 0.06 without augmentation to 0.84 +/- 0.02 using AugmentHE and HENorm. The results were statistically significant with p-values less than or equal to 0.0137 for all but one of the tested datasets. 

Model Structure

Outside of sampling and training data manipulations, some papers use their own special tools to mitigate bias. In an effort to classify skin lesions, Le et al. defines a focal loss equation, which is a function of the model’s confidence in its prediction [10]. The focal loss is a product of terms, including (1-p) and -log(p), where p is the probability the algorithm believes the sample is the actual correct value. Since these terms are large for small values of p, low confidence answers by the algorithm will heavily increase the focal loss, forcing the model to pay more attention to those samples. Using a model informed by focal loss, the authors showed that accuracy, precision, and F1 score increased from 0.74 to 0.90, 0.70 to 0.81, and 0.72 to 0.80, respectively, although no p-values or confidence intervals were provided, limiting the applicability of the results. Stanley et al. aims to reduce AI bias in diagnosing MRI brain images [11]. The paper theorized that a minority population’s characteristics may factor into the algorithm’s decision and lead to bias. They use another author’s “unlearning” tool that adjusts weights such that the model cannot predict the minority population but is still able to predict disease status from its metadata, and retrains the model iteratively from that point until accuracy plateaus. As the model was considered to have baseline performance on a certain area of the brain denoted "No Bias," the metrics used were relative change in true positive rate and false positive rate, with the former given by (Relative ΔTPR_a_) = ΔTPR_a_ - ΔTPRNo Bias, where ΔTPR_a_ is the difference in the TPR between the biased and non-biased group in the brain region "a" and ΔTPR_No Bias_ is defined analogously. With this tool, the relative change in true positive rate was reduced to 12.22 +/- 3.04% from 22.12 +/- 2.72% for predictions made in a certain part of the brain. There was improvement in the other two parts of the brain as well, and all results were statistically significant, but no p-values were provided.

To predict COVID diagnoses from hospital labs and data, Yang et al. uses the Kolmogorov-Smirnov test to see whether a given population differs from the majority, then uses a Markov decision process containing an “agent” variable that rewards correct sample classification [12]. One fairness metric used was equalized odds, defined as being achieved by a predictor if its P(Y'=1 | Y=y, Z=0) = P(Y'=1 | Y=y, Z=1) for y=0 and y=1, where Y' is the predicted label, Y is the actual label, and Z is an indicator variable for a feature. If we were to have y=0 correspond to the false positive rate, then y=1 would correspond to the true positive rate. In Yang et al's model, true positive and false positive equalized odds decreased to 0.056 and 0.204 from 0.070 and 0.226, respectively. Isensee et al. aims to recognize brain tumors on MRI and subsequently predict survival [13]. They approach the issue of having relatively few tumor examples in the training set by formulating a multiclass Dice loss function to minimize. The function measures the degree of pixel overlap between the actual tumor and the model’s identification of the tumor. High values of the loss function occur when there is low pixel overlap, suggesting that the model did not have good recognition of the tumor.

Cavallaro et al. aims to predict antimicrobial drug resistance, given a patient and hospital characteristics [14]. They use gradient-boosted decision trees that aim to predict the probability that a bacterium is resistant to a particular drug. Each independent variable used by the model to make its prediction contributes a “Shapley” value that is used to compute the log-odds log(p/1-p). The predictive power of a given independent variable is then ascertained from the Shapley values, where positive Shapley values indicate that the independent variable has greater contributions to the prediction and thus can be thought to represent more predictive power. The areas under receiver-operator curve for four common antibiotics were 0.78 (0.76, 0.81), 0.87 (0.85, 0.89), 0.99 (0.98, 0.99), and 0.79 (0.75, 0.81), where 95% confidence intervals are given in brackets. These high areas indicate strong model performance. Barda et al. uses another author’s “nudging” algorithm that iteratively adjusts a model’s predictions on a set of protected variables such that the predictions on those variables all fall within a predetermined limit of each other [15]. The algorithm was applied to two models, named PCE and FRAX, which predict cardiovascular disease and osteoporotic fractures, respectively, based on patient risk factors. One of their performance metrics, calibration-in-the-large (CITL), improved from 1.247 (1.227, 1.277) to 1.039 (1.025, 1.058) in the PCE and from 1.076 (1.065, 1.089) to 1.001 (0.992, 1.012) in the FRAX model, where CITL is "the difference between the mean observed and the mean predicted risk, expressed as an odds ratio," and 95% confidence intervals are given in brackets. A CITL of 1 indicates optimal performance, and larger values imply less optimal performance.

Evidence Appraisal and Clinical Readiness

The methodological maturity of the included studies varied substantially. Several studies demonstrated bias mitigation primarily in controlled, retrospective, benchmark, or simulated settings and were therefore considered preliminary technical evidence despite improvements in model performance or fairness metrics. This distinction was particularly important for studies in which bias mitigation was demonstrated using a single dataset, small minority classes, synthetic data, or simulated bias conditions. Stronger evidence was observed in studies that incorporated independent datasets, multicenter populations, or explicit out-of-sample validation. Benčević et al. evaluated skin color-associated performance both in- and out-of-sample across three datasets, while Franchet et al. trained pathology models using data from one cohort and assessed performance using images from other centers [9,17]. Yang et al. evaluated bias mitigation using data from hospitals in both high-income and low- and middle-income settings, and Kiyasseh et al. evaluated surgical AI across geographically distinct hospitals [12,18]. Barda et al. provided particularly strong evidence for subgroup calibration by evaluating PCE and FRAX predictions in cohorts exceeding one million patients and explicitly assessing calibration across intersecting demographic and socioeconomic subpopulations [15]. External validation was less common than internal validation, and several studies evaluated class imbalance rather than fairness across clinically relevant demographic subgroups. Reporting of missing data handling and subgroup-specific sample sizes was inconsistent. Calibration was also evaluated less frequently than discrimination or classification performance, despite its importance for clinical risk prediction. Consequently, improvement in accuracy, AUROC, sensitivity, specificity, or a fairness metric should not alone be interpreted as evidence that a bias-mitigation strategy is clinically generalizable. None of the included studies provided sufficient evidence to classify the evaluated mitigation strategy as fully validated through prospective real-world clinical implementation with longitudinal fairness monitoring. Thus, the available evidence predominantly supports technical feasibility and retrospective clinical validation rather than established clinical effectiveness.

The literature demonstrates that effective bias mitigation requires coordinated interventions spanning data preparation, model training, validation, and deployment. The included papers focus on key sources of bias at these stages of AI development and present corresponding mitigation techniques, which are summarized in Table 4. The contents of Table 4 will be defined and expanded on in the discussion section.

**Table 4:** Bias mitigation across the clinical AI lifecycle

Stage	Bias source	Mitigation
Data acquisition	Underrepresentation of patient subgroups	Broader recruitment, multi-institutional data, targeted sampling
Data preprocessing	Class imbalance, feature imbalance, missingness	Reweighting, oversampling, undersampling, augmentation
Model training	Learned representation bias	Fairness aware loss functions, adversarial learning, hybrid models
Validation	Unequal subgroup performance or calibration	Subgroup metrics, calibration assessment, external validation
Deployment	Dataset drift, clinician misuse, workflow mismatch	Monitoring systems, periodic recalibration, clinician oversight

Discussion

Identifying Bias

Before model training, it is important to identify subpopulations that may be at risk of disparate model performance. Dakshit et al. provides a basic framework for identifying such variables by considering the ratio of samples in a subpopulation to the samples from the majority population. For instance, if four patients in a dataset are of ethnicity A and the majority ethnicity of the dataset has 70 patients, then the authors define the bias score for ethnicity A to be 4/70. It follows that low bias scores indicate a low relative number of samples from the corresponding subpopulation and thus the model may perform less accurately on that subpopulation [16]. However, this method does not factor in the nature of the model, hence a low bias score does not guarantee poor model performance, and models may still perform poorly on subpopulations without a low bias score due to reasons other than underrepresentation.

Sample size must also be considered at the subgroup level rather than solely at the overall cohort level. A large overall dataset may provide limited evidence regarding fairness when relatively few observations or outcome events arise from the subgroup of interest. Conversely, resampling techniques may increase the apparent representation of a minority group without increasing the number of independent patients from whom information is available, potentially increasing overfitting. Accordingly, evaluations of bias mitigation should report both total sample size and, when available, the number of observations and outcome events within clinically relevant subgroups.

Sampling Methods

The literature on AI bias mitigation in medicine is most concrete in the areas of model development, training, and validation, with most ideas focusing on the training datasets or the structure of the model. Many of the discussed articles focus on bias as defined by weaker model performance on a certain subpopulation. The issue arises often due to there being fewer samples of the subpopulation, which we define as underrepresentation in Table 3. Of course, this may be addressed by synthesizing new samples from that subpopulation or by augmenting the dataset. This approach is frequently used when models are tasked with classifying pictures, as evidenced by StyleGAN and AugmentHE discussed earlier [8,9]. These programs create new images by modifying existing ones and including them in the training dataset. However, such programs are often limited to the situations they were designed for and difficult to create from scratch. Consequently, at the level of data acquisition, many authors focus on sampling methods. 

When considering sampling methods, Heidari et al’s approach involved downsampling the male majority class so that the male and female counts were similar and removed outliers for training defined by a tolerance of three standard deviations [4]. Approaches that define and exclude outliers are relatively easy to implement, but caution must be taken to ensure these outliers do not unknowingly constitute a subpopulation. This may especially be a risk if the samples are not normally distributed with respect to a given variable. For instance, if age were to follow a bimodal distribution in a population, then removing outliers based on distance to the mean would remove a significant number of samples. To avoid this issue, authors may compare sample characteristics between the outlier samples and the rest of the population. The distribution of sample characteristics should be considered when defining outliers, and authors may also set a percentage or absolute limit of samples that may be defined as outliers so as to avoid removing too many samples. 

One of the main limitations of undersampling is that information is lost when certain samples are excluded. Brown et al. uses the straightforward “normalized-cut” sampling, which simply ensures that the majority and minority samples are equal in number [6]. However, the ensemble outlier filtering method discussed in Brown et al’s paper is strong, as it effectively undersamples without losing information. They use another author’s ensemble outlier filtering method, which cleverly partitions the majority data into subsets T_i_ that are the same size as the minority subset T and trains one model on each T_i_ U T, ensuring all majority data are used while still giving the minority data equal consideration [6]. On the other hand, oversampling from a subpopulation may lead to overfitting. This is a particular risk with Li et al’s resampling by proportion, where only positive samples from the minority population are resampled [7]. That is, in an effort to eliminate bias against the variable of black race, only black patients with cardiovascular disease are resampled as opposed to all black patients. If a model was attempting to correctly identify samples with a disease where the disease prevalence is already low, then the number of samples that are both positive for the disease and also of the minority population may be incredibly low and such a resampling method may end up overfitting to those handful of samples. Therefore, evaluations of bias mitigation should report both total sample size and, when available, the number of observations and outcome events within clinically relevant subgroups.

One may also consider both undersampling the majority and oversampling the minority, but the black box nature of many models makes it difficult to predict the effect of multiple mitigation techniques. Consequently, it is recommended to run multiple models that use different combinations of mitigation techniques to find the best one. For instance, Zhao et al. evaluated models using undersampling, oversampling, and a combination of both (RUSBoost combined with AdaBoost) to observe which exhibited the best performance [5]. However, in general, it cannot be assumed that more techniques will result in more bias mitigated, as the techniques may interact unexpectedly. Similarly, attempting to mitigate bias across too many different groups may lead to overfitting and reduce generalizability. Barda et al. discuss an algorithm where authors specify subpopulations that they wish to reduce bias against, and for each subpopulation, the algorithm will iteratively compute the difference between its prediction and the true value and add the difference to its previous prediction if the difference exceeds an author-specified tolerance parameter. It will continue until the model’s predictions on all subpopulations are within those limits. Although the algorithm proved successful in the author’s paper, forcing a model’s performance to satisfy constraints across multiple variables may come at the cost of performance against each variable individually [15]. Authors must decide which variables are most important to address in their particular situation.

Multiple Datasets

The use of multiple datasets usually increases generalizability, but authors must decide how to compose both training and verification datasets. It is advisable to test many options, as was perfromed by Benčević et al. [17]. The authors combine three different datasets in two approaches: for the “out-sample” approach, they train the model on two of the datasets and test the model on the last dataset. In the “in-sample” approach, the model is trained and tested on data consisting of samples from all three datasets. The in-sample approach uses all datasets in both training and testing, so if the testing dataset has the same relative contributions from each individual dataset as the training dataset, then the testing data will be representative of the training, and good model performance supports model internal validity. However, the authors explain that since models hope to be useful outside the training data, it makes sense to subsequently use an out-sample approach and leave an entire dataset out of training to be used solely for testing. While this may support external validity, it means that any information in the excluded dataset will not be incorporated into the model, and the loss of information may diminish real world performance. This effect may depend on the sizes of the datasets. Authors may consider leaving each dataset out of the training data in turn and evaluating the performance of each such model.

Class and Feature Imbalance

For a model attempting to predict a certain outcome, we define class imbalance in Table 3 as a relative lack of samples with that outcome. For example, Isensee et al’s model aimed to identify brain tumors on MRI, and the lack of MRIs with brain tumors constitutes class imbalance [13]. This is distinct from underrepresentation bias, which could hypothetically constitute a lack of MRIs from a subpopulation such as race or ethnicity. This is similar but yet still distinct from feature imbalance, which occurs when an independent variable, or feature, is used to inform a model’s prediction, but there is a relative lack of samples with that feature. For instance, if the presence of diabetes in ventilated patients was used to inform a model’s prediction of patient mortality, then a lack of patients with diabetes compared with patients without diabetes would be an example of feature imbalance. However if the predictor variable was race or ethnicity and a dataset had a relatively low number of samples from a particular race or ethnicity, then the feature imbalance and underrepresentation bias for that race or ethnicity would be referring to the same phenomenon.

In many cases, however, class and feature imbalance are thought of as stemming from a lack of samples, so the sampling methods discussed previously could be used to alleviate these biases. However, other authors use reweighing to tackle class and feature imbalance, where disadvantaged samples receive some type of extra input or “weight” in the model. Examples include the focal loss equation or Dice loss function used by Le et al. and Isensee et al., which were discussed earlier [10,13]. Another notable example is by Kiyasseh et al., where an AI system named SAIS was used to identify which parts of a video of a surgery were most important in evaluating the surgeon’s skill [18]. They designed a neural network named TWIX that would augment SAIS decisions based on annotations by human evaluators. This example is notable, as it provides an instance of clinical human expertise influencing an otherwise “black box” AI model.

Adversarial Learning

Even after mitigating biases at the data acquisition and preprocessing stages, models can still exhibit weaker performances in the real world simply because the real-world scenario often differs from the training environment. No training dataset will be a perfect representation of the real world and population characteristics change with time. One technique to tackle this issue of representation bias in Table 3 is adversarial learning, as shown by Moskalenko et al. [19]. They introduce a training algorithm that takes existing training data and creates a perturbation from it, such as an adversarial attack or fault injection, that is sent into the model to increase the model’s familiarity with such “attacks.” The principle is that the perturbations can mimic the imperfect or inaccurate data the model may encounter in the real world. The authors did succeed in showing that adversarial learning increases fault tolerance for a model trained on medical datasets, but the technique was not directly applied to answer a real-world clinical question. Furthermore, the perturbations introduced may not be representative of the perturbations in real-world data. Consequently, the generalizability of their findings remains limited.

Medical Imaging

There are also specific biases and concerns pertaining to AI in medical imaging. The same training data biases discussed earlier still must be addressed, and Drukker et al. agrees that sampling methods and synthetic data can be very effective [20]. However, synthetic data itself must be generated from real data, which itself may contain biases that propagate into the synthetic images. Combining datasets may prove problematic if two datasets have the same data with different labels, which may occur in radiology or pathology. Ideally, enough reviewers are available to label training data to minimize errors. Whether or not the same CT scanners were used to generate the training data may affect model accuracy if applied to images generated by different CT scanners in the real world [20]. Multiple rounds of data analysis, label verification, and data labeling are recommended even before employing the specific techniques discussed in this paper. 

Issues continue in how AI is used by human reviewers. In the field of radiology, Ueda et al. points out that incorrect AI advice harmed mammogram reading performance across all radiologist expertise levels, and that inexperienced radiologists are more likely to follow incorrect AI [21]. In converse, even experienced radiologists may dismiss valid AI warnings due to alert fatigue if AI programs flag too many false positives. These phenomena reinforce the importance of educating human users on the characteristics and target demographic of an AI program. Human users must be aware what data the program is trained on and be familiar with any quirks or weaknesses. They also must be aware of the differences between the training data and the population the model is being used on. Fathi et al. explains how AI in imaging often has trouble with artifacts or overlying structures such as catheters or skin folds [22]. Obvious fractures are categorized as "doubt fractures" when the anatomy, patient position, or presence of other devices are slightly skewed. Given the wide range of factors that can affect AI use in imaging, Ueda et al. suggests a committee or department with the express goal of analyzing an AI program for evolving biases [21]. Such a department would track population drift, AI use by the hospital and medical professionals, performance discrepancies between subpopulations, and more. 

Missing Data

Missing data represent an additional potential source of algorithmic bias. Missingness may differ systematically across institutions or patient subgroups because of differences in healthcare access, diagnostic testing, documentation practices, or clinical workflows. Complete-case exclusion or imputation may therefore affect subgroups differently. Studies evaluating fairness should report the extent and pattern of missingness, the methods used to address missing values, and whether missingness differs across protected or clinically relevant subgroups. In the present evidence appraisal, missing data handling was recorded as not reported when sufficient information could not be identified, rather than assuming missingness was absent.

Performance Metrics

It is often advisable to use multiple metrics for evaluating bias. For instance, Benčević et al. uses three different metrics for bias on the subject of identifying skin lesions, with two focusing on the boundary of the lesion and the last measuring overall sensitivity and precision [17]. This addresses the fact that their model may perform differently on different parts of a skin lesion and thus offers a more complete quantification of bias. The use of multiple metrics is also useful as not all metrics are clinically applicable. Many papers use sensitivity and specificity as metrics, which are commonly used clinical metrics, but do not always capture how far off a model is from the ground truth. On the other hand, metrics such as mean squared error capture distance well but do not necessarily inform clinical practice. On this note, it is essential to conduct satisfactory analysis to decide whether a given bias mitigation technique leads to a statistically, and ideally, clinically significant increase in performance. For instance, a model with a statistically significant increase in predictive accuracy compared with a previously used method may not be clinically useful if the model is comparatively far more time-consuming or difficult to implement than the previously used method. The lack of such significance does not imply a given method is hopeless, as it can always be improved upon in the future or applied to new situations.

Discrimination and calibration should also be considered separately when assessing clinical readiness. Measures such as AUROC, sensitivity, and specificity quantify aspects of discrimination but do not establish that predicted probabilities correspond to observed clinical risk. A model may demonstrate strong discrimination while systematically overestimating or underestimating risk within a particular subgroup. Barda et al. illustrates the importance of this distinction by explicitly evaluating CITL and calibration across intersecting subpopulations [15]. Therefore, studies reporting improvements in discrimination or fairness metrics without corresponding calibration assessment should be interpreted cautiously when considering clinical implementation.

Proposed Protocol to Mitigate Bias in Clinical AI Application Development

Based on our findings, the following proposed protocol outlines strategies to mitigate bias during the development of clinical AI models. Principle 1: Bias mitigation must occur across multiple stages of development. Principle 2: Single-technique approaches are insufficient. Principle 3: Fairness-performance tradeoffs are unavoidable. Principle 4: External validation is essential for fairness generalizability. Principle 5: Human oversight remains necessary even in debiased systems. Principle 6: Technical fairness does not establish clinical readiness.

It has been demonstrated that bias can occur at any stage, with the included papers focusing on the stages of data preprocessing, model training, and validation in Table 3. Consequently, multiple approaches tackling bias at each stage are indicated. However, fairness interventions may introduce performance tradeoffs, where optimizing equity metrics across protected populations can occasionally reduce overall predictive accuracy or calibration, necessitating careful balancing of fairness and clinical utility according to the intended use case. For instance, a tradeoff between overall accuracy and optimization of equalized odds or equalized opportunity in a model aiming to predict disease must take disease prevalence and clinician utility into account, as those performance metrics are informed by false positive and true positive rates. The utility in optimizing these rates between subpopulations over general accuracy may be lessened for severe diseases such as cancer where the diagnosis should never be missed. On the other hand, achieving equality across subpopulations at the expense of general accuracy may be desirable for diseases that are otherwise harder to diagnose in a disadvantaged subpopulation. Some skin conditions are more commonly misdiagnosed on dark skin, upon which a predictive model aimed at this target population may favor optimizing equalized odds to improve performance on this population at the expense of the majority population, possibly leading to decreased overall accuracy. Therefore, authors must consider how they wish their model to be used and what the target populations are, alongside a consideration of what biases to address and what tradeoffs to accept. It is recommended to use different combinations of techniques across multiple stages of development and to compare performances. Bias mitigation should progress through internal validation, independent external validation, prospective clinical evaluation, and post-deployment monitoring. At each stage, performance should be evaluated both overall and within clinically relevant subgroups, including discrimination, calibration, and clinically meaningful error rates. A mitigation strategy should not be considered clinically validated solely because it improves a fairness metric within the development dataset.

Strength of Evidence and Clinical Readiness

The findings of this review demonstrate an important distinction between successful bias mitigation in a development environment and clinical validation of a bias-mitigation strategy. Internal test set performance demonstrates that a method can reduce a specified measure of bias under the conditions represented by the development dataset. It does not establish that the same improvement will persist across institutions, demographic compositions, disease prevalence, imaging devices or other data acquisition equipment, and clinical workflows. External validation therefore represents an important transition in evidence maturity. Studies using geographically, institutionally, or otherwise independently derived datasets provide stronger evidence that mitigation effects generalize beyond the development environment. Nevertheless, external retrospective validation remains distinct from prospective clinical evaluation. A model that demonstrates equitable retrospective performance may still behave differently after deployment. For instance, population characteristics may "drift" over time, rendering models outdated and decreasing performance over years or decades. Clinician trust remains a critical determinant of adoption, as healthcare providers may be hesitant to integrate AI systems that function as “black boxes” or demonstrate inconsistent subgroup performance. These issues constitute the final source of bias in the deployment stage as observed in Table 3. Monitoring systems are therefore indicated to detect dataset drift, emerging subgroup disparities, and unintended clinical consequences that may arise over time. Even with such systems, however, real-world deployment remains limited by barriers, including heterogeneous electronic health record systems, inconsistent data quality, limited interoperability, and the computational and institutional resources required to continuously retrain and evaluate models across diverse patient populations. Accordingly, the evidence supporting the mitigation strategies identified in this review should be viewed as a continuum rather than as equivalent proof of clinical effectiveness. Most approaches currently demonstrate either technical feasibility or retrospective clinical validation. Prospective evaluation, assessment of patient or clinician outcomes, and longitudinal post-deployment fairness monitoring remain comparatively underdeveloped. This gap should be considered when translating apparently successful fairness interventions into clinical practice.

On this note, other reviews examining bias mitigation methods have also pointed out the relative lack of exploration into post-processing and post-deployment methodologies. Chen et al's review focusing on mitigation methods pertaining to electronic health records found only one article discussing post-processing methods [23]. Similar to our review, most papers they investigated used reweighing and resampling methods. This highlights post-processing and post-deployment methods as a continued gap in knowledge and an important target for future research.

Regulatory oversight is also becoming increasingly important as organizations such as the Food and Drug Administration and World Health Organization emphasize transparency, accountability, and ongoing safety evaluation for AI-enabled medical technologies [24,25]. Consequently, external validation across geographically, demographically, and institutionally distinct datasets is essential to ensure that bias mitigation strategies generalize beyond the original training environment. Ultimately, effective bias mitigation carries significant health equity implications, as poorly validated or inadequately monitored AI systems risk perpetuating existing disparities in diagnosis, treatment, and healthcare access among historically underserved populations.

This review has several limitations. First, it is a structured narrative review rather than a systematic review or meta-analysis, and studies published before 2018 were excluded to emphasize contemporary approaches. The search strategy may not have captured all synonymous terminology used to describe bias and fairness mitigation. Second, substantial heterogeneity in clinical tasks, datasets, model architectures, definitions of bias, and performance metrics precluded quantitative comparison or meta-analysis. Third, reporting of subgroup sample sizes, missing data handling, calibration, and external validation was inconsistent across the included studies. Our evidence maturity categories therefore represent a structured descriptive appraisal rather than a validated quantitative risk-of-bias instrument. Finally, few studies prospectively evaluated mitigation strategies in clinical workflows, and evidence regarding longitudinal performance after deployment was particularly limited. Publication bias may also favor successful mitigation approaches.

## Conclusions

In the realm of healthcare AI, there have been many advancements in bias mitigation, with some key common themes. For composing a training dataset, authors have explored sampling methods and synthetic data to tackle underrepresentation. Beyond this, authors have devised specific improvements in existing algorithms or have come up with their own bias mitigation techniques to tackle their unique problem. Overall, bias mitigation in healthcare AI has advanced considerably, but the maturity of the supporting evidence varies substantially. Improvements in fairness or predictive performance within a retrospective development dataset should not be equated with clinical validation. Stronger evidence requires adequately powered subgroup analyses, transparent handling of missing data, assessment of subgroup-specific discrimination and calibration, independent external validation, and ultimately prospective evaluation in clinical workflows.

We hope our proposed protocol will encourage multifaceted approaches that address bias at all stages of development. Future directions in healthcare AI will increasingly focus on foundation models, multimodal systems, and clinician-AI collaboration to improve clinical decision-making and patient care. Emerging approaches such as federated learning and continuous fairness monitoring can support privacy-preserving, equitable model deployment across healthcare systems. At the same time, explainable and fair AI will remain essential for building clinician trust, ensuring transparency, and enabling safe integration of AI into real-world clinical workflows.
